# A randomized controlled comparison of non-channeled king vision, McGrath MAC video laryngoscope and Macintosh direct laryngoscope for nasotracheal intubation in patients with predicted difficult intubations

**DOI:** 10.1186/s12871-019-0838-z

**Published:** 2019-08-31

**Authors:** Haozhen Zhu, Jinxing Liu, Lulu Suo, Chi Zhou, Yu Sun, Hong Jiang

**Affiliations:** Department of Anesthesiology, Shanghai Ninth People’s Hospital Affiliated to Shanghai Jiao Tong University School of Medicine, 639 Zhizaoju Road, Shanghai, 200011 China

**Keywords:** Airway management - video laryngoscopes - Nasotracheal intubation

## Abstract

**Background:**

King Vision and McGrath MAC video laryngoscopes (VLs) are increasingly used. The purpose of this study was to evaluate the performance of nasotracheal intubation in patients with predicted difficult intubations using non-channeled King Vision VL, McGrath MAC VL or Macintosh laryngoscope by experienced intubators.

**Methods:**

Ninety nine ASA I or II adult patients, scheduled for oral maxillofacial surgeries with El-Ganzouri risk index 1–7 were enrolled. Patients were randomly allocated to intubate with one of three laryngoscopes (non-channeled King Vision, McGrath MAC and Macintosh). The intubators were experienced with more than 100 successful nasotracheal intubations using each device. The primary outcome was intubation time. The secondary outcomes included first success rate, time required for viewing the glottis, Cormack-Lehane grade of glottis view, the number of assist maneuvers, hemodynamic responses, the subjective evaluating of sensations of performances and associated complications.

**Results:**

The intubation time of King Vision and McGrath group was comparable (37.6 ± 7.3 s vs. 35.4 ± 8.8 s) and both were shorter than Macintosh group (46.8 ± 10.4 s, *p* < 0.001). Both King Vision and McGrath groups had a 100% first attempt success rate, significantly higher than Macintosh group (85%, *p* < 0.05). The laryngoscopy time was comparable between King Vision and McGrath group (16.7 ± 5.5 s vs. 15.6 ± 6.3 s) and was shorter than Macintosh group (22.8 ± 7.2 s, *p* < 0.05) also. Compared with Macintosh laryngoscope, Glottis view was obviously improved when exposed with either non-channeled King Vision or McGrath MAC VL (*p* < 0.001), and assist maneuvers required were reduced (*p* < 0.001). The maximum fluctuations of MAP were significantly attenuated in VL groups (47.7 ± 12.5 mmHg and 45.1 ± 10.3 mmHg vs. 54.9 ± 10.2 mmHg, *p* < 0.05 and *p* < 0.01). Most device insertions were graded as excellent in McGrath group, followed by Macintosh and King Vision group (*p* = 0.0014). The tube advancements were easier in VLs compared with the Macintosh laryngoscope (*p* < 0.001). Sore throat was found more frequent in Macintosh group compared with King Vision group (*p* < 0.05).

**Conclusions:**

Non-channeled King Vision and McGrath MAC VLs were comparable and both devices facilitated nasotracheal intubation in managing predicted difficult intubations compared with Macintosh laryngoscope.

**Trial registration:**

ClinicalTrials registration number NCT03126344. Registered on April 24, 2017.

**Electronic supplementary material:**

The online version of this article (10.1186/s12871-019-0838-z) contains supplementary material, which is available to authorized users.

## Introduction

The video laryngoscope (VL) has been well established as an approach in airway management for patients with difficult direct laryngoscopy [1–5]. However, most of the literatures focused on their usage for oral intubation. Nasotracheal intubation (NTI), often required for oral and maxillofacial operation, may be complicated by causing injuries to the nasal passage and sinusitis [6]. In addition, a superior laryngoscopy does not guarantee a successful advancement of the tube into the trachea and external manipulation of the larynx, a Magill forceps, change in head position or partial inflation of cuff is required [6–9].

**Fig. 1:**
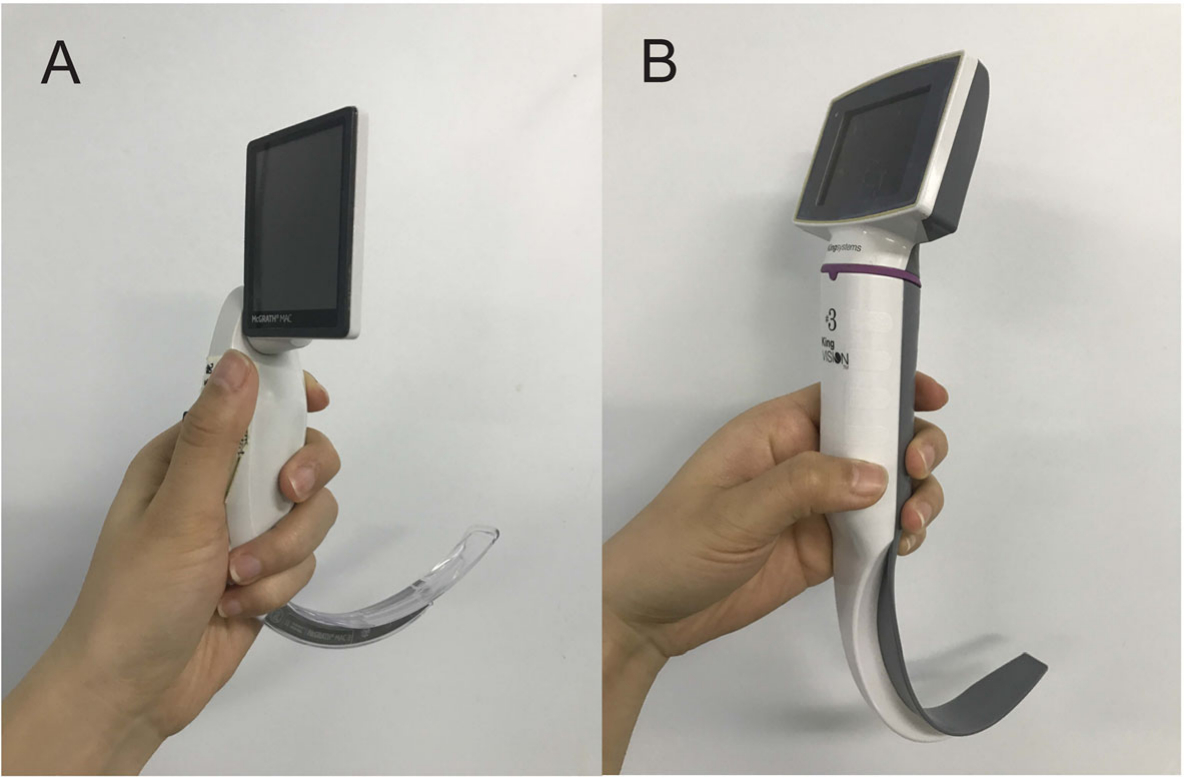
The video laryngoscopes evaluated in our study. A: non-channeled King Vision video laryngoscope. B: McGrath MAC video laryngoscope

The success of a VL assisted intubation depends on multiple factors, such as blade design (acute angled or Macintosh like; channeled or non-channeled); quality of the image on the monitor, as well as the experience of the intubator [5, 10]. Recently, the McGrath MAC VL (Fig. 1a) (Aircraft Medical, Edinburgh, UK) has been widely used. It has a battery powered handle, on the top of which is an adjustable liquid crystal display monitor. It has an angulated single-use blade without a guiding channel. It was reported to facilitate routine NTIs in normal patients compared with Macintosh laryngoscope [11, 12]. The King Vision VL (Fig. 1b) (Ambu Inc., Denmark) is also a portable device with similar design with McGrath MAC VL. King Vision VL is relative newer and cheaper. Different from McGrath, its monitor is fixed to the handle. It has a channel integrated to the blade to facilitate tube guidance into the trachea though, channeled King Vision VL required longer time and provided lower success rates on first attempt for oral intubation in normal airway compared to McGrath MAC VL [13]. It is argued that channeled devices are often bulky and can be difficult to use in patients with limited mouth opening [2, 13, 14]. However, the King Vision VL is also available with standard non-channeled blade for NTI. Recent study also found time for tracheal intubation could be shortened by using a non-channeled blade [15]. In addition, the King Vision VL is reported that can provide a better vision condition which may be beneficial to NTI.

A recent systematic review comparing VL versus DL for NTI showed that VL is particularly beneficial for patients with difficult airways [16]. However, only two randomized controlled trials (Airtriq and C-MAC versus Macintosh) were enrolled [7, 17]. It remains unclear whether non-channeled King Vision or McGrath MAC VL, compared with conventional laryngoscope, provide shorter intubation time and a higher first success rate for NTI when used by experienced provider in management of predicted difficult intubation. It is also unclear whether non-channeled King Vision VL is superior to McGrath MAC VL when used for NTI. We therefore performed this randomized, controlled trial to fill the gap. Our hypothesis was that the non-channeled King Vision and McGrath MAC VL were comparable, and both video devices were superior to Macintosh laryngoscope in terms of shorter intubation time and higher first success rate.

## Methods

### Ethics approval and consent to participate

This trial was approved by IRB (2017–308-T228) from Shanghai Ninth People’s Hospital Affiliated to Shanghai Jiao Tong University School of Medicine, and registered at clinicaltrials.gov (NCT03126344). Written consents to participate were obtained from all participants after enrollment. Our study was adhered to the applicable Consolidated Standards of Reporting Trials (CONSORT) guidelines.

### Subjects

Consecutive patients, between 18 and 60 years old with American Society of Anesthesiologists (ASA) classification of I or II, and requiring NTI for elective oral and maxillofacial surgery, were screened in the Preoperative Evaluation Unit of our institute. Prediction of difficult intubation is graded by El-Ganzouri multivariate risk index (EGRI) based on seven parameters (body weight, modified Mallampati class, mouth opening, thyromental distance, neck movement, prognathism, and history of difficult airway) [17, 18] (Additional file 1). Patients were enrolled if EGRI score 1–7 [17]. In cases where an awake NTI was planned [i.e. EGRI score > 7, history of reflux or diagnosed oesophageal disease, severe obstructive sleep apnea (OSA) and morbid obesity (body mass index > 40 kg/m^2^)] were excluded from the study.

### Method of anesthesia

The study was designed as a single blind, three parallel arms, randomized controlled trial comparing NTIs using non-channeled King Vision VL, McGrath MAC VL and Macintosh DL in adults with predictors of difficult airways. The size 3 blades were used in both King Vision and McGrath group. The standard Macintosh blade (size 3 for female; size 4 for male) was used as control.

Patients were asked which nostril was clearer. If both sides were equal and the surgeon had no objection, the right nostril was chosen [19]. Patients were randomly assigned to King Vision group, McGrath group or Macintosh group via a computer generated randomization table. All NTIs were performed by attending anesthesiologists experienced with more than 100 successful NTIs with each device.

No premedication was administered. Lactated Ringer’s solution infusion was started intravenously to deal with the fluid loss from the overnight fast after entering the operating theatre. A standard preparation was then performed, including heart rate (HR), lead II ECG, SpO_2_ (pulse oximetry), and end expiratory carbon dioxide. A Bispectral (BIS) index sensor was attached to the patient’s forehead in conjunction with the BIS Monitor. Cannulation of right radial artery was performed under local anesthesia for invasive blood pressure monitoring.

All patients were preoxygenated by a facemask in the position of neutral. Prior to anesthesia induction, the nasal mucosa was well prepared with 1% tetracaine hydrochloride jelly for 2 min and five drops of ephedrine hydrochloride nitrofurazone (containing approximately 2 mg ephedrine) in all patients. Baseline hemodynamic data were recorded by an investigator after a stabilization period of 10 min.

The nasotracheal tube used was reinforced endotracheal tube (ETT, Safety-Flex with Murphy Eye, oral/nasal, Athlone, Ireland; ID 6.5 mm in female and ID 7.0 mm in male patients) and was well lubricated with 1% tetracaine hydrochloride jelly. Dosing of induction medications was given at the discretion of the attending anesthesiologists. Induction agents included midazolam (0.02 mg/kg), propofol (1.5~2 mg/kg) and fentanyl (2 μg/kg). Upon loss of consciousness and jaw relaxation, manual ventilation was tested. If manual ventilation was available, cissatracurium besilate (0.15 mg/kg) was administrated and post induction values were recorded 3 min after induction. Unsuccessful manual ventilation led to study exclusion.

The anesthesiologist tried to intubate when the Train of Four (TOF) count reached zero and BIS value decreased to 50. NTI was performed in a standard manner. First, a preformed ETT was inserted into the nostril and advanced to the posterior nasopharyngeal wall. Second, a laryngoscope blade was introduced into the mouth to expose the glottis. If it’s necessary, the BURP maneuver (backward, upward, right-sided pressure) on the thyroid cartilage was attempted to obtain good glottis visibility [20]. And ultimately, the ETT was inserted into the trachea with the aid of Magill’s forceps, head flexion, or cuff inflation if necessary.

The primary outcome was the intubation time, defined as the interval between opening the mouth and the time when three consecutive end-tidal CO_2_ waves were appeared on the monitor. Since the time required for SpO_2_ to decrease during apnea was about 150 s [21], we defined a failure as the intubation time took longer than 150 s [22], SpO_2_ less than 92% or oesophagus intubation. The patient was mask ventilated after a failed attempt. In VL groups, the intubator should try the other video device for the second attempt. In Macintosh group, the patient’s airway was managed using either non-channeled King Vision or McGrath MAC VL at the discretion of the intubator. If the second attempt was still unsuccessful, the fiberoptic bronchoscope (FOB) was applied. If intubation is not possible with FOB, the patient was awakened.

Secondary outcome measures included time to expose the glottis (laryngoscopy time) and the view of glottis opening valued by Cormack-Lehane grade. A Cormack-Lehane grade IV was defined as laryngoscopy failure. A blinded investigator also recorded the hemodynamic changes (MAP, HR) during the procedure of NTI. The maximum values of invasive MAP were recorded. After successful intubation, the subjective sensation of the intubator (ease of device insertion, quality of view on display and ease of tube advancement) was graded as excellent, good, fair and poor. Other intubation parameters included incidences of bleeding or dental injury, number of assist maneuvers (use of BURP maneuver, Magill’s forceps, or cuff inflation). Twenty four hours after the procedure, a nurse anesthetist blinded to group assignment recorded the severity of sore throat and hoarseness.

### Statistical analysis

Our sample size estimation was based on previous studies [11, 23], in which the standard deviations (SD) of intubation time were estimated as 8 and 13.7 s. To detect a intergroup difference of 10 s in intubation time with α of 0.05 and β of 0.8, we estimated that 30 patients would be enough for each group. To compensate for patients dropping out during the study, additional patients (10%) were added. The final sample size of 33 patients was in each group.

Mean (SD) or Median (IQR [range]) was used to describe the parametric data. The number (percentage) was used to describe nonparametric data. Statistical analyses were performed with Prism 5.0 for Windows (GraphPad Software, Inc., La Jolla, California, USA). Binary data for three groups were analyzed using chi-square test and each two groups were compared with chi-square segmentation method or Fisher’s exact test as appropriate. One-way analysis of variance (ANOVA) with post-hoc Bonferroni’s Multiple Comparison test was used to analyze parameter data for changes within groups. The Kruskal-Wallis ANOVA with post-hoc Dunn’s test was used to analyze ordinal data. A *p* value less than 0.05 was considered as significant.

## Results

**Fig. 2:**
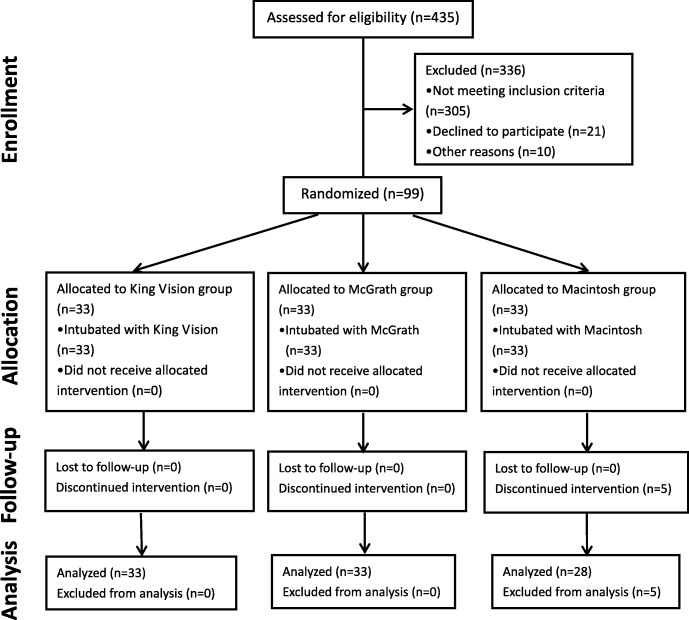
Consort flow chart

In total, 99 patients were enrolled in this study between June 2017 and January 2018(Fig. 2). The distribution of the patient characteristics and difficult intubation predictors were well balanced between three groups (Table 1). Five patients in Macintosh group were intubated successfully with VLs (two patients with King Vision and three patients with McGrath VL) after failed intubation attempt. All failures were due to poor glottis exposure and esophagus intubation. These patients were excluded from follow-up data analysis as these outcomes were not controlled.

**Table 1:** Patient characteristics and difficult intubation profiles

	King Vision *n* = 33	McGrath *n* = 33	Macintosh *n* = 33	*p* value
Men (%)	15 (45%)	19 (58%)	16 (48%)	NS
Age; years	38 (12)	36 (11)	40 (11)	NS
BMI; Kg·m^−2^	22 (3)	22 (3)	22 (3)	NS
ASA class I/II (%)	11/22 (33/67%)	15/18 (45/55%)	13/20 (40/60%)	NS
Neck movement < 80° (%)	2 (6%)	1 (3%)	1 (3%)	NS
Mallampati III or IV (%)	30 (91%)	31 (94%)	30 (91%)	NS
Interincisor gap < 3 cm (%)	9 (27%)	10 (30%)	8 (27%)	NS
Thyromental distance < 6 cm (%)	8 (27%)	9 (27%)	11 (33%)	NS
Ability to prognath (%)	30 (91%)	28 (85%)	29 (88%)	NS
EGRI scores	3 (2,4.5)	3 (2.5,4)	3 (2,4)	NS

Data presented as mean (SD), median (IQR [range]) or number of patients (percentage). BMI: Body Mass Index. ASA class: American Society of Anesthesiologists classification. EGRI: El-Ganzouri risk index. NS: not significant

Regarding the primary outcome measure intubation time, King Vision and McGrath groups were comparable (37.6 ± 7.3 s vs. 35.4 ± 8.8 s) and both were significantly shorter than Macintosh group (46.8 ± 10.4 s, *p* < 0.001, Table 2).

**Table 2:** Intubation profiles

	King Vision *n* = 33	McGrath *n* = 33	Macintosh *n* = 33	*p* value
Intubation time (sec) ^1^	37.6 (7.3) ***	35.4 (8.8) ***	46.8 (10.4)	< 0.0001
First success of intubation (%)	33 (100%) *	33 (100%) *	28 (85%)	0.0017
Laryngoscopy time (sec) ^1^	16.7 (5.5) *	15.6 (6.3) *	22.8 (7.2)	0.0002
C-L grade I/II/III/IV	29/4/0/0 ***	27/6/0/0 ***	6/10/12/5	< 0.0001
C-L grade I and II (%)	33 (100%) ***	33 (100%) ***	16 (48%)	0.0004
Assist maneuvers (%) ^1^	5 (15%) ***	4 (12%) ***	18 (64%)	< 0.0001
Difference between MAP_maximum_ and MAP _post-induction_ (mmHg) ^1^	47.7 (12.5)*	45.1 (10.3)**	54.9 (10.2)	0.0030

Data presented as mean (SD) or number (percentage). C-L: Cormack and Lehane. MAP: mean arterial pressure. NS: not significant. Assist maneuvers: use of the BURP maneuver, Magill’s forceps or cuff inflation. ^1^: n = 28 in Macintosh group. *p < 0.05; **p < 0.01; ***p < 0.001 compared with Macintosh group

Regarding the secondary outcomes, both King Vision and McGrath groups had a 100% first attempt success rate, significantly higher than Macintosh group (85%, *p* < 0.05, Table 2). The laryngoscopy time was comparable between King Vision and McGrath groups (16.7 ± 5.5 s vs. 15.6 ± 6.3 s) and were significantly shorter than Macintosh group (22.8 ± 7.2 s, *p* < 0.05, Table 2), also. Glottis view was obviously improved when exposed with either non-channeled King Vision or McGrath MAC VL: the percentage of Cormack-Lehane grade I or II was 100% in VLs groups and 48% in Macintosh group, respectively (*p* = 0.0004, Table 2). The number of assist maneuvers required was 5, 4 and 18 in King Vision, McGrath and Macintosh group, respectively (*p* < 0.0001, Table 2). The SpO_2_ did not differ between groups.

**Fig. 3:**
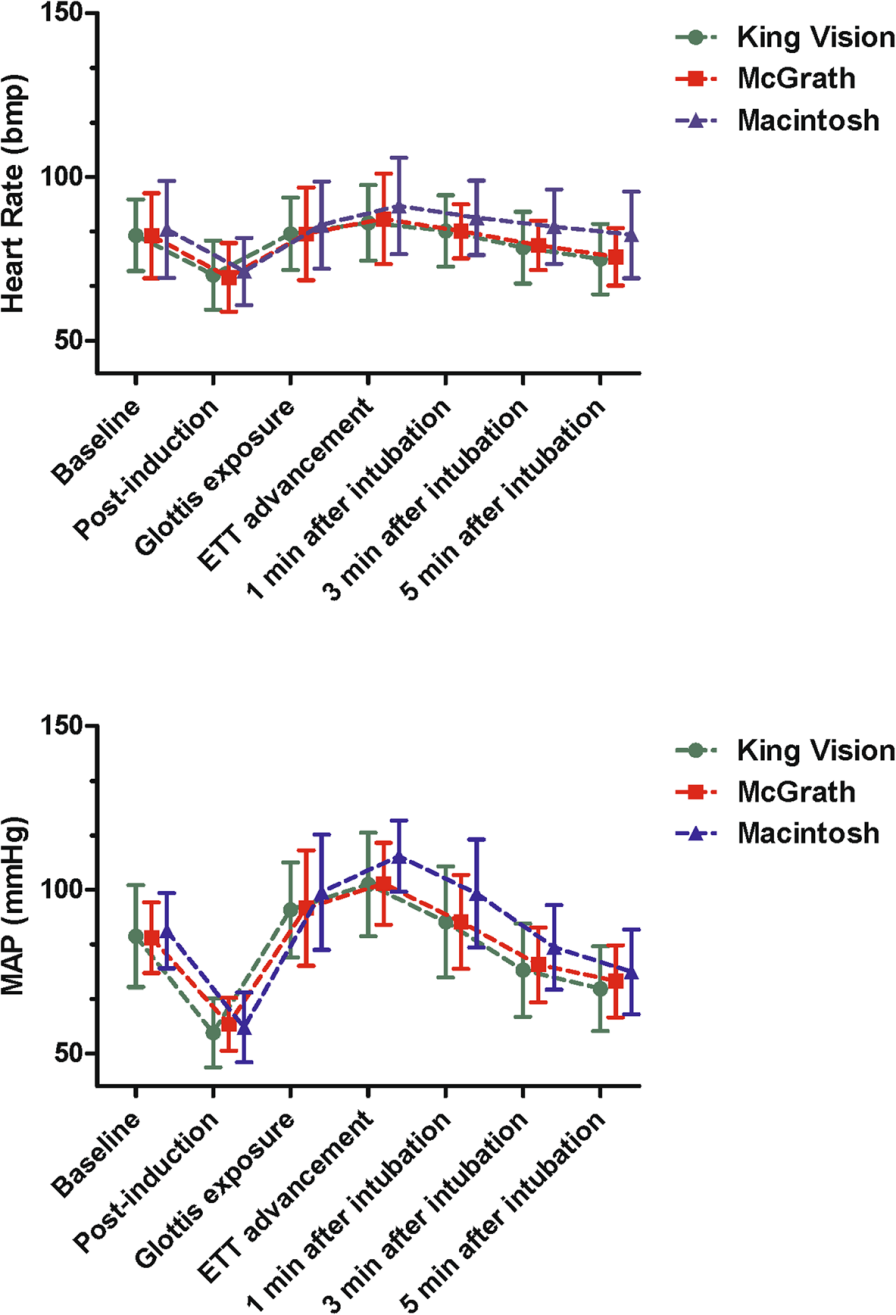
Changes in hemodynamic responses during anesthesia induction and intubation. Up: Heart Rate. Down: MAP. MAP: mean arterial pressure

Changes in hemodynamic responses during anesthesia induction and intubation were demonstrated in Fig. 3. Briefly, both MAP and HR decreased significantly in each group after anesthesia induction. Then glottis exposure with each laryngoscope and following ETT placement caused significantly increase in MAP and HR. Finally, MAP and HR descended slowly 1, 3, 5 min after successful intubation. Notably, the maximum fluctuations of MAP (MAP_max_ − MAP_post-induction_) in King Vision and McGrath groups were comparable and both were significantly attenuated compared with Macintosh group (47.7 ± 12.5 mmHg and 45.1 ± 10.3 mmHg vs. 54.9 ± 10.2 mmHg, *p* < 0.05 and *p* < 0.01 respectively, Table 2).

Results of the subjective sensations between devices were listed in Table 3. Most device insertions were graded as excellent in McGrath group (91%), followed by Macintosh (82%) and King Vision group (54%) (*p* = 0.0014). Quality of view on display did not differ between King Vision and McGrath groups. The ease of tube advancement was comparable between King Vision and McGrath groups, and both were much better than Macintosh group (*p* < 0.001). There were no cases of desaturation and dental injury during NTIs. Sore throat was found more frequent in Macintosh group compared with King Vision group (*p* < 0.01). Occurrence of bleeding and hoarseness seemed more frequent in Macintosh group, but failed to show significance. These symptoms were minor and ceased spontaneously without intervention.

**Table 3:** Sensations of performances and any complications

	King Vision *n* = 33	McGrath *n* = 33	Macintosh *n* = 28	*p* value
Ease of device insertion (excellent/good/fair/poor)	18/13/2/0 # (54/40/6/0%)	30/3/0/0 (91/9/0/0%)	23/5/0/0 (82/18/0/0%)	0.0014
Quality of view on display (excellent/good/fair/poor)	33/0/0/0 (100/0/0/0%)	32/1/0/0 (97/3/0/0%)	/	NS
Ease of tube advancement (excellent/good/fair/poor)	28/5/0/0 *** (85/15/0/0%)	29/4/0/0 *** (88/12/0/0%)	13/10/4/1 (46/36/14/4%)	0.0001
Desaturation (%)	0 (0)	0 (0)	0 (0)	NS
Bleeding (%)	1 (3%)	0 (0)	4 (14%)	0.0357
Dental injury (%)	0 (0)	0 (0)	0 (0)	NS
Sore throat (%)	3 (11%) **	8 (24%)	12 (43%)	0.0093
Hoarseness (%)	2 (6%)	1 (3%)	5 (18%)	0.969

Data presented as number (percentage). NS: not significant. #*p* < 0.05 compared with McGrath group. *p < 0.05; **p < 0.01; ***p < 0.001 compared with Macintosh group

## Discussion

Although many studies about indirect laryngoscopes were carried out, only two randomized controlled trial, to our knowledge, have compared the VLs (Airtraq and C MAC respectively) with Macintosh laryngoscope in patients showing predictors of difficult nasal intubation [7, 17]. King Vison and McGrath MAC VLs are relative newer and are also well worth studying. We provided the first study about the non-channeled King Vision and McGrath MAC VLs for NTIs in predicted difficult patients.

Our main result was that the time to successful NTI with both VLs was significantly faster than with Macintosh DL. The intubation time mainly comprises two parts: time to view the vocal cords and time required for tube passage through glottis. Firstly, we confirmed both non-channeled King Vision and McGrath MAC VL significantly shortened the laryngoscopy time in predicted difficult patients, which was certainly a reason for a shortened intubation time. The result showed less association between predictors of difficult intubations and glottis exposure using non-channeled King Vision or McGrath MAC VL than using Macintosh DL. Secondly, laryngoscopy with non-channeled King Vision or McGrath MAC caused less anterior elevation of the larynx than invasive direct laryngoscopy because airway axes alignment was not needed. This might provide a more direct route from nasopharynx to glottis and therefore ease advancing the tube into the trachea. In current study, we confirmed it was easier to advance the ETT through the glottis, accordingly less frequency of assist maneuvers was required in VL groups. Thirdly, when doing oral intubation with VLs, we often bend the styletted tracheal tube to a greater degree (‘hockey stick’ like) to follow the curvature of the video blade, which always hinder stylet removal and increase intubation time [24, 25]. However, stylet was not required for NTI in our study. So use of VLs also saved time required for tube advancement by decreasing the frequency of additional assist maneuvers and stylet removal [7, 11, 24]. We noticed the laryngoscopy time was longer in King Vision group compared with McGrath group. Alvis et al., in a recent comparison with McGrath, suggested that it was difficult when inserting the channeled blade of King Vision VL into the mouth [13]. Here we found it also more difficult when introducing the non-channeled King Vision blade compared with McGrath blade. The blade of King Vision is longer and more acute angled. We agree with the author who claimed a specific angle to the patient’s chest was required when insertion King Vision ‘L’ shaped blade [13]. On the contrary, the blade design of the McGrath VL is similar to the classic Macintosh DL. This provides the intubator with a familiar laryngoscopy experience. The channeled blade of King Vision may decrease the oral cavity for tube adjustment and advancement during oral intubation [2, 13]. During NTI, however, no such difficulty was observed when advancing the trachea tube with non-channeled King Vision VL. This was in contrast to oral intubation [13]. Although the intubation time was a little bit longer in King Vision group, the clinical relevant is debatable because SpO2 was not different between King Vision and McGrath VL.

The predictors of difficult airways used in our study are reliable [26]. To compare difficult intubation levels, EGRI was used in our study [17, 18]. It is reported that EGRI > 7 were more suitable for awake fiberoptic intubation [27]. Therefore, only patients with EGRI score 1–7 were included in our study. Although the variation of enrollment was big, the EGRI scores were similar between groups. In addition, the distributions of risk factor were also comparable. The proportion of Cormark-Lehane grade III and IV in Macintosh group suggested patients enrolled were predicted difficult intubations. The 15% failure rate of Macintosh DL seem quite high though, the success rate of Macintosh DL (85%) was similar to what has been described previously [3, 17].

Both non-channeled King Vision and McGrath MAC VLs improved the Cormack-Lehane grade significantly which was the main superiority of VLs. St Mont et al. demonstrated first attempt success rate of NTI by Airtraq was 94% in predicted difficult airway [7]. Hazarika H et al. reported a 98% first attempt success rate of NTI by C-MAC D-Blade VL for difficult nasal intubation [17]. Here, we demonstrated the success rate of first attempt of NTI was 100% in non-channeled King Vision and McGrath groups. The ‘you see that you fail’ situations of King Vision reported previously [2, 14] did not occur in present study. This could be explained by the fact that its ‘L’ shape blade conformed to the upper airway well though; it always hindered oral ETT advancement. While during NTI, the shape and size of the non-channeled King Vision blade has little influence on the tube advancement and oral cavity allowed for nasotracheal tube adjustment is big enough. Therefore, these results clearly demonstrate that both non-channeled King Vision and McGrath MAC VLs are good choices for NTIs. All failed Macintosh assisted NTIs were because of the poor glottis view, even with the help of assist maneuvers. These patients were eventually easily intubated on the first attempt with either non-channeled King Vision or McGrath MAC VLs. We believe that both of them can serve as a promising backup alternative for failed NTI using Macintosh DL. However, VLs are not the Holy Grail [28]. Actually, VLs will fail under certain conditions; the total success range was 37–98% in literature [2]. Although our results seem to suggest a 100% success rate of VLs intubation, our result should be interpreted with caution due to small sample size. Also, the results of this study may not be applicable to other types of patients, such as severe OSA or morbid obesity. The NTIs in our study were done by experienced attending anesthesiologists. Hence, there may generalize bias in experience.

To avoid missing any hemodynamic response, invasive blood pressure was used in current study. In addition, the maximum fluctuation of MAP was chosen to reflect the hemodynamic change. This could partially explain why our data were different from previous study [17]. During NTI, stimulations of the nasopharyngeal structures, oropharyngeal structures and trachea induced by laryngoscopy or ETT advancement are three main stages of hemodynamic changes [29]. To optimizing glottis exposure in difficult laryngoscopy patients, enhanced upward lifting force of Macintosh blade was required [30]. The laryngeal prominence was excessively compressed and the oropharynx structure was therefore distorted. In such circumstance, assist maneuvers were often used to help the ETT through the glottis in the Macintosh group. However, the VLs allow to view glottis from the monitor, intubate tube using less maneuvers and potentially less force which minimized stimuli applied to the oropharyngeal structures during intubation [31]. Our data strongly demonstrated that the non-channeled King Vision and McGrath MAC VLs might provide clinical advantages in attenuating the hemodynamic changes to potential difficult NTI patients.

Most participants felt McGrath blade insertions were easiest. That was because of its slim design as we discussed before. McGrath MAC was lightest and more portable than the others. The monitor could be adjusted to an optimized angle for intubation. Although it was claimed that the King Vision VL could provide a better vision condition, we did not see the difference. Quality of view on display did not differ between King Vision and McGrath VLs. Reducing the usage of assist maneuvers, fewer demanding of the physical workload and lower anterior pressure exerted on the soft structures could be linked to reduced sore throat and hoarseness occurrences in both VL groups. The King Vision group had the fewest cases of sore throat. We guessed the length and angle of the King Vision non-channeled blade might be more beneficial to exposure of glottis compared with Macintosh like blade, and accordingly less workload was required. However, oral surgical procedures might confound these results since they tend to cause similar symptoms, and further study is required.

Some limitations of our study should be considered carefully. First, neither the intubator nor the independent observer could be blinded from the groups. However, we have minimized adverse effects by defining robust outcome measures. Second, if we compared both Cormark-Lehane and POGO scores, our results should be more convincing. Third, our results might be biased by the variable experience of the intubator with different laryngoscope. It is believed that intubation with VL require a complex hand-eye-coordination competencies which grow with a learning curve [32]. On the other hand, it was suggested that novices could translate direct laryngoscopy technique to video laryngoscopy if it is similar to classic Macintosh laryngoscope [33]. Therefore, the results might not necessarily be obtained by novice users. Finally, the participants included did not represent genuine difficult airways. We believe it ethically questionable to test a new intubation device on genuine difficult airway patients. Therefore, further studies may be carried out to clarify these issues.

## Conclusion

In summary, we observed that NTIs with non-channeled King Vision and McGrath VLs in the setting of predicted difficult intubations resulted in shorter intubation time, higher first success rate, better qualities of glottis view, attenuated hemodynamic responses, and fewer incidences of side effect compared with Macintosh DL. These data provided evidence that NTI using King Vision and McGrath were comparable, and both devices were superior to Macintosh DL in managing the difficult intubations.

## Additional files

Additional file 1: El-Ganzouri risk index. (DOC 44 kb)

## Data Availability

The datasets of current study are available from the corresponding author on reasonable request.

## Abbreviations

ASA: America Society of Anesthesiologists
BMI: Body Mass Index
DL: Direct laryngoscope;
EGRI: El-Ganzouri risk index
HR: Heart rate
MAP: Mean arterial pressure
NTI: Nasotracheal intubation
VL: Video larynogoscope

## References

[1] Lewis SR, Butler AR, Parker J, et al. Videolaryngoscopy versus direct laryngoscopy for adult patients requiring tracheal intubation. Cochrane Database Syst Rev. 2016;11:CD011136. 10.1002/14651858.CD011136.pub2 PMC6472630 27844477

[2] Kleine-Brueggeney M, Greif R, Schoettker P (2016). Evaluation of six videolaryngoscopes in 720 patients with a simulated difficult airway: a multicentre randomized controlled trial. Br J Anaesth.

[3] Aziz MF, Dillman D, Fu R, Brambrink AM (2012). Comparative effectiveness of the C-MAC video laryngoscope versus direct laryngoscopy in the setting of the predicted difficult airway. Anesthesiology.

[4] Malik MA, Subramaniam R, Maharaj CH (2009). Randomized controlled trial of the Pentax AWS, Glidescope, and Macintosh laryngoscopes in predicted difficult intubation. Br J Anaesth.

[5] Pieters BMA, Maas EHA, Knape JTA, van Zundert AAJ (2017). Videolaryngoscopy vs. direct laryngoscopy use by experienced anaesthetists in patients with known difficult airways: a systematic review and meta-analysis. Anaesthesia.

[6] Hall CE, Shutt LE (2003). Nasotracheal intubation for head and neck surgery. Anaesthesia.

[7] St Mont G, Biesler I, Pfortner R (2012). Easy and difficult nasal intubation--a randomised comparison of Macintosh vs Airtraq(R) laryngoscopes. Anaesthesia.

[8] Staar S, Biesler I, Muller D (2013). Nasotracheal intubation with three indirect laryngoscopes assisted by standard or modified Magill forceps. Anaesthesia.

[9] Kumar R, Gupta E, Kumar S (2013). Cuff inflation-supplemented laryngoscope-guided nasal intubation: a comparison of three endotracheal tubes. Anesth Analg.

[10] Huitink JM, Bouwman RA (2015). The myth of the difficult airway: airway management revisited. Anaesthesia.

[11] Kwak HJ, Lee SY, Lee SY (2016). McGrath video laryngoscopy facilitates routine Nasotracheal intubation in patients undergoing Oral and maxillofacial surgery: a comparison with Macintosh laryngoscopy. J Oral Maxillofac Surg.

[12] Sato Boku A, Sobue K, Kako E (2017). The usefulness of the McGrath MAC laryngoscope in comparison with Airwayscope and Macintosh laryngoscope during routine nasotracheal intubation: a randomaized controlled trial. BMC Anesthesiol.

[13] Alvis BD, Hester D, Watson D (2016). Randomized controlled trial comparing the McGrath MAC video laryngoscope with the king vision video laryngoscope in adult patients. Minerva Anestesiol.

[14] Kleine-Brueggeney M, Buttenberg M, Greif R (2016). Evaluation of three unchannelled videolaryngoscopes and the Macintosh laryngoscope in patients with a simulated difficult airway: a randomised, controlled trial. Anaesthesia.

[15] Kriege M, Alflen C, Noppens RR (2017). Using king vision video laryngoscope with a channeled blade prolongs time for tracheal intubation in different training levels, compared to non-channeled blade. PLoS One.

[16] Jiang J, Ma DX, Li B (2019). Videolaryngoscopy versus direct laryngoscopy for nasotracheal intubation: a systematic review and meta-analysis of randomised controlled trials. J Clin Anesth.

[17] Hazarika H, Saxena A, Meshram P, Kumar Bhargava A (2018). A randomized controlled trial comparing C mac D blade and Macintosh laryngoscope for nasotracheal intubation in patients undergoing surgeries for head and neck cancer. Saudi J Anaesth.

[18] el-Ganzouri AR, McCarthy RJ, Tuman KJ (1996). Preoperative airway assessment: predictive value of a multivariate risk index. Anesth Analg.

[19] Seo KS, Kim JH, Yang SM (2007). A new technique to reduce epistaxis and enhance navigability during nasotracheal intubation. Anesth Analg.

[20] Takahata O, Kubota M, Mamiya K (1997). The efficacy of the "BURP" maneuver during a difficult laryngoscopy. Anesth Analg.

[21] Eichhorn L, Erdfelder F, Kessler F (2017). Influence of apnea-induced hypoxia on catecholamine release and cardiovascular dynamics. Int J Sports Med.

[22] Jones PM, Armstrong KP, Armstrong PM (2008). A comparison of glidescope videolaryngoscopy to direct laryngoscopy for nasotracheal intubation. Anesth Analg.

[23] Bamgbade OA, Onaolapo MH, Zuokumor PA (2011). Nasotracheal intubation with the McGrath videolaryngoscope in patients with difficult airway. Eur J Anaesthesiol.

[24] Taylor AM, Peck M, Launcelott S (2013). The McGrath(R) series 5 videolaryngoscope vs the Macintosh laryngoscope: a randomised, controlled trial in patients with a simulated difficult airway. Anaesthesia.

[25] Jones PM, Turkstra TP, Armstrong KP (2007). Effect of stylet angulation and endotracheal tube camber on time to intubation with the GlideScope. Can J Anaesth.

[26] Eberhart LH, Arndt C, Aust HJ (2010). A simplified risk score to predict difficult intubation: development and prospective evaluation in 3763 patients. Eur J Anaesthesiol.

[27] Cortellazzi P, Minati L, Falcone C (2007). Predictive value of the El-Ganzouri multivariate risk index for difficult tracheal intubation: a comparison of Glidescope videolaryngoscopy and conventional Macintosh laryngoscopy. Br J Anaesth.

[28] Sgalambro F, Sorbello M (2017). Videolaryngoscopy and the search for the holy grail. Br J Anaesth.

[29] Singh S, Smith JE (2003). Cardiovascular changes after the three stages of nasotracheal intubation. Br J Anaesth.

[30] Carassiti M, Zanzonico R, Cecchini S (2012). Force and pressure distribution using Macintosh and GlideScope laryngoscopes in normal and difficult airways: a manikin study. Br J Anaesth.

[31] Gaszynski T, Jakubiak J (2016). Muscle activity during endotracheal intubation using 4 laryngoscopes (Macintosh laryngoscope, Intubrite, TruView Evo2 and king vision) - a comparative study. Med Pr.

[32] Cortellazzi P, Caldiroli D, Byrne A (2015). Defining and developing expertise in tracheal intubation using a GlideScope((R)) for anaesthetists with expertise in Macintosh direct laryngoscopy: an in-vivo longitudinal study. Anaesthesia.

[33] Herbstreit F, Fassbender P, Haberl H (2011). Learning endotracheal intubation using a novel videolaryngoscope improves intubation skills of medical students. Anesth Analg.

