# Supplementary material for: A randomized controlled comparison of non-channeled king vision, McGrath MAC video laryngoscope and Macintosh direct laryngoscope for nasotracheal intubation in patients with predicted difficult intubations
Source: BMC Anesthesiol. 2019 Aug 31;19:166. doi: 10.1186/s12871-019-0838-z (PMC6717380; doi:10.1186/s12871-019-0838-z)
Supplement: Supplementary file 1 — El-Ganzouri risk index. (DOC 44 kb) [file 12871_2019_838_MOESM1_ESM.doc]

**Appendix: El-Ganzouri risk index**

| Variable | Points |
| --- | --- |
| Mouth opening |  |
| >= 4 cm | 0 |
| < 4 cm | 1 |
| Thyromental distance |  |
| > 6.5 cm | 0 |
| 6.0-6.5cm | 1 |
| < 6.0 cm | 2 |
| Mallampati class |  |
| I  II  III | 0  1  2 |
| Neck movement  > 90° | 0 |
| 80-90° | 1 |
| < 80° | 2 |
| Ability to prognath |  |
| Yes | 0 |
| No | 1 |
| Body weight | 0 |
| < 90 kg | 0 |
| 90-100 kg | 1 |
| > 100 kg | 2 |
| History of difficult intubation |  |
| None | 0 |
| Questionable | 1 |
| Definite | 2 |
